# A Position Statement on the Utility of Interval Imaging in Standard of Care Brain Tumour Management: Defining the Evidence Gap and Opportunities for Future Research

**DOI:** 10.3389/fonc.2021.620070

**Published:** 2021-02-09

**Authors:** Thomas C. Booth, Gerard Thompson, Helen Bulbeck, Florien Boele, Craig Buckley, Jorge Cardoso, Liane Dos Santos Canas, David Jenkinson, Keyoumars Ashkan, Jack Kreindler, Nicky Huskens, Aysha Luis, Catherine McBain, Samantha J. Mills, Marc Modat, Nick Morley, Caroline Murphy, Sebastian Ourselin, Mark Pennington, James Powell, David Summers, Adam D. Waldman, Colin Watts, Matthew Williams, Robin Grant, Michael D. Jenkinson

**Affiliations:** ^1^School of Biomedical Engineering & Imaging Sciences, King’s College London, London, United Kingdom; ^2^Department of Neuroradiology, King’s College Hospital NHS Foundation Trust, London, United Kingdom; ^3^Centre for Clinical Brain Sciences, University of Edinburgh, Edinburgh, United Kingdom; ^4^Brainstrust, Cowes, United Kingdom; ^5^Leeds Institute of Medical Research at St James’s, St James’s University Hospital, Leeds, United Kingdom; ^6^Faculty of Medicine and Health, Leeds Institute of Health Sciences, University of Leeds, Leeds, United Kingdom; ^7^Siemens Healthineers, Frimley, United Kingdom; ^8^The Brain Tumour Charity, Fleet, United Kingdom; ^9^Department of Neurosurgery, King’s College Hospital NHS Foundation Trust, London, United Kingdom; ^10^ACT for Cancer, London, United Kingdom; ^11^The Tessa Jowell Brain Cancer Mission, London, United Kingdom; ^12^Lysholm Department of Neuroradiology, National Hospital for Neurology and Neurosurgery, London, United Kingdom; ^13^Department of Oncology, Christie Hospital NHS Foundation Trust, Manchester, United Kingdom; ^14^Department of Neuroradiology, The Walton Centre NHS Foundation Trust, Liverpool, United Kingdom; ^15^Department of Radiology, Wales Research and Diagnostic PET Imaging Centre, Cardiff University School of Medicine, Cardiff, United Kingdom; ^16^King’s College Trials Unit, King’s College London, London, United Kingdom; ^17^King’s Health Economics, King’s College London, London, United Kingdom; ^18^Department of Oncology, Velindre Cancer Centre, Cardiff, United Kingdom; ^19^Department of Neuroradiology, Western General Hospital, Edinburgh, United Kingdom; ^20^Birmingham Brain Cancer Program, University of Birmingham, Birmingham, United Kingdom; ^21^University Hospitals Birmingham NHS Foundation Trust, Birmingham, United Kingdom; ^22^Department of Neuro-oncology, Imperial College Healthcare NHS Trust, London, United Kingdom; ^23^Institute of Translational Medicine, University of Liverpool, Liverpool, United Kingdom; ^24^Department of Neurosurgery, The Walton Centre NHS Foundation Trust, Liverpool, United Kingdom

**Keywords:** glioblastoma, high grade glioma, glioma, meningioma, interval imaging, magnetic resonance imaging, utility, monitoring biomarker

## Abstract

**Objectiv e:**

To summarise current evidence for the utility of interval imaging in monitoring disease in adult brain tumours, and to develop a position for future evidence gathering while incorporating the application of data science and health economics.

**Methods:**

Experts in ‘interval imaging’ (imaging at pre-planned time-points to assess tumour status); data science; health economics, trial management of adult brain tumours, and patient representatives convened in London, UK. The current evidence on the use of interval imaging for monitoring brain tumours was reviewed. To improve the evidence that interval imaging has a role in disease management, we discussed specific themes of data science, health economics, statistical considerations, patient and carer perspectives, and multi-centre study design. Suggestions for future studies aimed at filling knowledge gaps were discussed.

**Results:**

Meningioma and glioma were identified as priorities for interval imaging utility analysis. The “monitoring biomarkers” most commonly used in adult brain tumour patients were standard structural MRI features. Interval imaging was commonly scheduled to provide reported imaging prior to planned, regular clinic visits. There is limited evidence relating interval imaging in the absence of clinical deterioration to management change that alters morbidity, mortality, quality of life, or resource use. Progression-free survival is confounded as an outcome measure when using structural MRI in glioma. Uncertainty from imaging causes distress for some patients and their caregivers, while for others it provides an important indicator of disease activity. Any study design that changes imaging regimens should consider the potential for influencing current or planned therapeutic trials, ensure that opportunity costs are measured, and capture indirect benefits and added value.

**Conclusion:**

Evidence for the value, and therefore utility, of regular interval imaging is currently lacking. Ongoing collaborative efforts will improve trial design and generate the evidence to optimise monitoring imaging biomarkers in standard of care brain tumour management.

## Introduction

Over the last decade the treatment landscape for adult brain tumours has changed incrementally for some tumour types, such as metastases, where there have been improvements in systemic therapy and brain radiotherapy (1). For other tumour types there has been little change. The management of glioblastoma remains largely based on maximum safe resection and radiotherapy with concomitant and adjuvant temozolomide chemotherapy (2). Evidence from randomised controlled trials [level 1 (3)] underpins clinical treatments of adult brain tumours. In contrast, there is little evidence (< level 3) to support the current imaging practices used to monitor disease progression or response to treatment (4, 5). Therefore, the clinical utility [the relevance and usefulness of an intervention in patient care using all sources of evidence (6)] of interval imaging (imaging at pre-planned time-points to assess tumour status, as compared with scanning for reasons of clinical deterioration) is largely unknown.

“Interval imaging” was first introduced into neuro-oncology in 1977 by Victor Levin, shortly after the introduction of computed tomography (CT) (7). In 1981 the World Health Organisation (WHO) convened two expert meetings on the “Standardization of Reporting Results of Cancer Treatment”. The recommendations were widely adopted to ensure consistency of timing between centres and became the basis of subsequent iterations of high-grade glioma treatment monitoring and result reporting (8). These evolved with the development of MRI. In 1990, Macdonald also recommended assessing factors affecting imaging appearance such as corticosteroid use (9) which subsequently formed the basis of the AVAglio trial response criteria (10) and the 2010 response assessment in neuro-oncology (RANO) trial guidelines (11). There is a demonstrable historical lineage following the advent of CT for how enhancing tumour size, following exogenous contrast administration, has been incorporated into current clinical and trial practice. The expert committees were informed by observational studies, supported by a biologically plausible assumption that the images from each time-point, or the rate of change in a series of imaging investigations, are reliable “monitoring biomarkers” (12) reflecting tumour behaviour. The assumption is that changes in tumour size identify progression of disease, potentially before it becomes clinically apparent, resulting in a lead time improvement for therapeutic intervention. Indeed, there may be benefits in changing management before the development of irreversible disability or before the extent of tumour precludes intervention. Some justification for enhancement as a disease proxy has been inferred from data showing that enhancing tumour size and extent of resection are “prognostic biomarkers” (12) at both first presentation and recurrence (13–15). However, there is no evidence that earlier diagnosis influences prognosis pre- or post-operatively, and individual enhancing tumour growth trajectories vary between individuals with the same histological tumour type.

Deriving an evidence base surrounding current imaging practices is important for several reasons. There is a lack of biological specificity for contrast enhancement, particularly in the context of treatment effects and pseudophenomena, which can confound imaging assessment. There is also variability in clinical adoption of interval imaging practice across UK and European neuro-oncology centres which is unlikely to be in the best interests of patients or healthcare systems (16, 17). It is also noteworthy that the widely reported observation that increasing enhancement tracks growth of most brain tumour types, has extended the use of contrast-enhancing tumour size as a biomarker beyond high-grade glioma. The impetus to derive an evidence-base is driven by researchers (4, 5, 16), but more importantly patients and carers (**Box 1**) (18). This in part relates to factors such as understanding the anxiety surrounding the imaging event and awaiting the results – so-called “scanxiety” (19). Furthermore, determining the health economics related to interval imaging is equally important. These include understanding the direct costs of subsequent investigations (for example, ‘advanced imaging’ requiring additional sequences and processing time, or earlier interval imaging than usual), additional hospital appointments that may follow uncertain tumour changes, continuation of futile therapies, as well as indirect and opportunity costs.

The purpose of this position statement is to summarise the current evidence base for the utility of interval imaging in brain tumours and to propose potential studies for future evidence gathering incorporating the disciplines of data science and health economics.

Box 1 **A National Institute for Health Research (NIHR) James Lind Alliance (JLA) Priority.**

The National Institute for Health Research (NIHR) James Lind Alliance (JLA) brings patients, carers and clinicians together in Priority Setting Partnerships stating that “addressing uncertainties about the effects of a treatment should become accepted as a routine part of clinical practice” and that “patients, carers and clinicians should work together to agree which, among those uncertainties, matter most and deserve priority attention”.

In 2015, the group met to establish the ten highest clinical priority uncertainties in neuro-oncology in the UK. Number two is: “what is the effect on prognosis of interval scanning to detect tumour recurrence compared with scanning on symptomatic recurrence in people with a brain tumour?” Patients expect imaging will give an accurate account of the effect of treatment and either reassure or initiate a change in the treatment plan. If there is uncertainty regarding progression, that leads to anxiety until the next scan or specialist MR imaging. In addition, if there is minor imaging progression only, there may be the clinical dilemma as to whether to change management if there are further options, even though the patient has had no clinical deterioration and it is not known whether earlier pre-symptomatic intervention improves survival.

This might be interpreted as:

“For me, a patient, does earlier scanning to detect asymptomatic progression improve my quality of life and survival, or does it not make any difference, or make it worse?”

And:

“Would I, as a doctor, improve the quality of life and survival of my patients if I monitored them more proactively and detected progression before it became symptomatic or does it not make any difference or make it worse?”

## Materials And Methods

Clinicians, scientists, and patient advocates and representatives with expertise in interval imaging, data science, health economics or trial management of adult brain tumours, convened in London, UK, in April 2019 in conjunction with a National Cancer Research Institute (NCRI) Brain Tumour group workshop. Available evidence for interval imaging pathways for different tumour types was discussed in the context of research recommendations of previous publications, including the UK’s National Institute for Health and Care Excellence (NICE) brain tumour guidelines NG99 (**Supplementary Table 1**) and a systematic review of glioma imaging (4, 5, 16). Specifically, we sought to assess value in the context of morbidity, mortality, quality of life and resource use (together these outcomes give the additional outcome measure of cost effectiveness). Clinical utility incorporates all these outcome measures as well as considering the interests and goals of stakeholders (6). In addition, the results of a UK national clinical practice survey on the use of internal imaging in glioblastoma management were reviewed. Opportunities to generate evidence were explored in the context of specific study designs, with a focus on the utility and limitations of applying each design to a specific scenario. We outlined the advantages and disadvantages of each design based on current evidence and expert opinion. The discussion was compiled into a manuscript and circulated to NCRI Brain Tumour Group members and invited experts in attendance as well as those unable to attend. Edits and feedback were incorporated until all authors were in agreement with the content, and a position statement was produced around potential approaches to studying interval imaging in glioma and meningioma.

## Results

### Targeting of Interval Imaging Studies

Following explicit agreement that there was an evidence gap, and that the JLA priority (**Box 1**) should be addressed, an initial question was to determine which brain tumour types should be included in the position statement. Whilst central nervous system (CNS) tumours comprise a range of diverse histological and molecular entities (20), meningioma and gliomas are the two commonest accounting for 36% and 28% of tumours, respectively. These were selected as the focus for interval imaging studies. Although brain metastases are more common overall, because of the rapidly evolving treatment paradigms according to primary cancer site (stereotactic radiosurgery, chemotherapy, immunotherapy) (1), the reliance on disease response assessment in the body to systemic treatments, and the fact that this can even vary between different intracranial lesions in the same individual (1), it was agreed that metastases were beyond the scope of the workshop (21–23). It was acknowledged that high-grade (WHO III-IV) and lower-grade (WHO II) infiltrating gliomas should be treated separately. Individual imaging biomarker techniques beyond standard structural clinical MRI have been reviewed extensively elsewhere (24, 25) and the uptake of these “advanced” MRI and positron emission tomography (PET) techniques is limited and highly heterogeneous, even in specialist neuro-oncology centres across Europe (17). The focus at the workshop was therefore on determining the value of the structural MRI interval imaging pathway and methods to interrogate this, although the potential role of additional imaging techniques remains relevant. Improving the diagnostic performance of structural or “advanced” imaging biomarkers is a further means to rationalise imaging timepoints by reducing repeat imaging while there is ongoing uncertainty. Key points regarding study design types are summarised in **Table 1**.

**Table 1:** Different study designs to interrogate interval imaging.

Design	Time direction	Tumour and Comment	Objectives and Comment	Advantages	Disadvantages
Audit (single centre)	Retrospective	HGG Meningioma LGG	• understand MDTM decision making (not morbidity and mortality) • understand if there is standardised MRI interval imaging protocol or not; if so protocol details	• fast way to estimate factors influencing MDTM decision making • can inform more complex studies • some modelling of health economic outcomes plausible in meningioma/LGG who undergo imaging over many years	• level 3 evidence (confounded data capture) • high likelihood that modelling of health economic outcomes may fail
Audit (multicentre)	Retrospective	HGG Meningioma LGG	• understand MDTM decision making (not morbidity and mortality) • modelling of health economic outcomes; requires some data to predict what would have happened in the absence of the change in management (counterfactual outcomes). • understand if there is standardised MRI interval imaging protocol or not; if so protocol details	• larger numbers: more statistical inference • more representative sample • fast way to estimate factors influencing MDTM decision making • modelling of health economic outcomes plausible in meningioma/LGG who undergo imaging over many years • can inform more complex studies	• level 3 evidence (confounded data capture) • time consuming preparing data collection • effort could be going into level 1 or 2 evidence • robustness in the modelling of health economic outcomes dependent on quality of data used to extrapolate counterfactual outcomes
Observational (single centre)	Prospective	HGG Meningioma LGG	• test decision making at MDTM (not morbidity and mortality) • No need to examine those imaging timepoints clearly changing management if audit shows this already • understand if there is standardised MRI interval imaging protocol or not; if so protocol details	• level 2 evidence • accurately understand all factors influencing MDTM decision making • potential to capture data on multiple timepoints throughout pathway	• time consuming collecting prospective data • maybe challenging for a health economic assessment given a lack of comparator data may limit the scope of economic evaluation
Observational (multicentre) (additional points relating to cancer registries shown in bold)	Prospective	HGG Meningioma LGG	• test decision making at MDTM (not morbidity and mortality) • no need to examine those imaging time points clearly changing management if audit shows this already • understand if there is standardised MRI interval imaging protocol or not; if so protocol details • **cancer registries may allow high level interval imaging comparisons**	• level 2 evidence • accurately understand all factors influencing MDTM decision making • potential to capture data on multiple timepoints throughout pathway • more representative sample • possibly RADIANT (UK) perform study • **quality assured available resource** • **with large datasets, interval imaging comparisons, accounting for health service organization variation, may provide useful information**	• time consuming preparing data collection • time consuming collecting prospective data • **data may not be detailed enough & incomparable**
RCT (multicentre)	Prospective	HGG Meningioma & LGG challenging given long follow up	• compare: symptomatic imaging vs. regular interval imaging • non-inferiority study	• level 1 evidence to assess morbidity, mortality • analysis of quality of life & resource use outcomes using trial data and extrapolation with a decision model • Answers JLA question	• recruitment challenging both for patients & PI (“no equipoise”). This may be explored by feasibility study. • time consuming preparing data collection • time consuming collecting data • expensive
RCT (multicentre)	Prospective	HGG	• compare: EPMRI vs. No EPMRI • non-inferiority study	• level 1 evidence to assess morbidity, mortality • analysis of quality of life & resource use outcomes using trial data and extrapolation with a decision model	• recruitment challenging both for patients & PI (“no equipoise”). This may be explored by feasibility study. • time consuming preparing data collection • time consuming collecting data • expensive
RCT (multicentre)	Prospective	HGG Meningioma & LGG challenging given long follow up	• compare: joint decision between patient and clinician for imaging vs. regular interval imaging • non-inferiority study	• level 1 evidence to assess morbidity, mortality • analysis of quality of life & resource use outcomes using trial data and extrapolation with a decision model	• recruitment challenging both for patients & PI (“no equipoise”). This may be explored by feasibility study. • time consuming preparing data collection • time consuming collecting data • expensive
RCT (multicentre)	Prospective	HGG Meningioma & LGG challenging given long follow up	• compare: imaging vs. no imaging at a timepoint informed by prior studies • non-inferiority study	• level 1 evidence to assess morbidity, mortality • analysis of quality of life & resource use outcomes using trial data and extrapolation with a decision model	• recruitment challenging both for patients & PI (“no equipoise”). This may be explored by feasibility study. • time consuming preparing data collection • time consuming collecting data expensive
RCT (multicentre)	Prospective	HGG Meningioma & LGG challenging given long follow up	• compare: imaging vs no imaging at multiple (or all) timepoints • non-inferiority study	• level 1 evidence • to assess morbidity, mortality analysis of quality of life & resource use outcomes using trial data and extrapolation with a decision model • Analysis of multiple points of current pathway	• recruitment challenging both for patients & PI (“no equipoise”). This may be explored by feasibility study. • potential ethical concerns • complex • very large numbers required as multiple decision points • time consuming preparing data collection • time consuming collecting data expensive
RCT (multicentre)	Prospective	HGG Meningioma & LGG challenging given long follow up	• compare: imaging at multiple short interval timepoints (e.g. at 1 month) vs routine interval imaging (e.g. at 3 months) • non-inferiority study	• level 1 evidence • to assess morbidity, mortality • analysis of quality of life & resource use outcomes using trial data and extrapolation with a decision model • Optimal way to understand points of progression	• compliance • challenging both for patients & PI. This may be explored by feasibility study. • difficult to justify increased scans if no clear step towards a change in management after scans. • time consuming preparing data collection • time consuming collecting data very expensive
*in silico* (single centre)	Retrospective	HGG Meningioma LGG	• discover high value imaging time points • understand influence of co-variates	• some discovery prediction • some modelling opportunities • some limited ability to inform RCTs of best imaging point to analyse • some ability to look at different levels of granularity including radiomics • some assessment of morbidity, mortality • may support some model based economic evaluation • small possibility of finding ways to improve survival	• lower level evidence (level not quantifiable) • (confounded data capture) • modelling limited with small numbers
*in silico* (multicentre)	Retrospective	HGG Meningioma LGG	• discover high value imaging time points • understand influence of co-variates	• larger numbers: more discovery prediction • more representative sample • modelling opportunities ability to look at different levels of granularity including radiomics • inform RCTs of best imaging point to analyse • leverage additional trial data e.g. EORTC or intellance AbbVie (2 month follow up) or Paradigm (3 month follow up) or CODAGLIO trial data • more accurate modelling of morbidity, mortality, • may support some model based economic evaluation • possibility of finding ways to improve survival	• lower level evidence (level not quantifiable) (confounded data capture) • time consuming preparing data collection
*in silico* (single centre)	Prospective	HGG Meningioma & LGG challenging given long follow up	• discover high value imaging time points • understand influence of co-variates	• Moderately higher level evidence (level not quantifiable) • some discovery prediction • some modelling opportunities inform RCTs of best imaging point to analyse • ability to look at different levels of granularity including radiomics • potential to add MR fingerprinting • moderately more accurate modelling of morbidity, mortality, quality of life, resource use, co-variates • small possibility of finding ways to improve survival	• modelling limited with very small numbers • time consuming collecting data • if adding additional experiments e.g. MR fingerprinting, trial more complex
*in silico* (multicentre) Non-federated (additional federated points shown in bold)	Prospective	HGG Meningioma & LGG challenging given long follow up	• discover high value imaging time points • understand influence of co-variates	• Higher level evidence (level not quantifiable) • larger numbers: more discovery prediction • more representative sample • modelling opportunities • inform RCTs of best imaging point to analyse • ability to look at different levels of granularity including radiomics • potential to add MR fingerprinting • leverage Health Data Research UK BRAIN MATRIX, BRIAN • more accurate modelling of morbidity, mortality, quality of life, resource use co-variates • possibility of finding ways to improve survival • **overcome concerns regarding de-identification** • **constant iteration of models**	• time consuming preparing data collection • time consuming collecting data expensive • if adding additional experiments e.g. MR fingerprinting, trial more complex • **federated learning is still at the research stage: time consuming to develop and resolve challenges**

Different study designs can be used individually, although a combination of studies will likely be necessary to address different issues around interval imaging impacts.

### Interval Imaging Overview

Neuro-oncology multi-disciplinary team meetings (MDTs, referred to as “Tumor Boards” in North America) consider longitudinal patient management, with serial imaging follow-up having a central role. Meningioma and glioma imaging follow up schedules from UK’s National Institute for Health and Care Excellence (NICE) brain tumour guidelines NG99 are shown in **Supplementary Tables 2** and **3** (4). In the broadest sense, interval imaging is typically performed in order to determine whether a tumour is growing, which may initiate or change treatment. A planned imaging schedule provides clinicians with a framework to track an aspect of tumour biology just before clinical review and allows easier administrative timetabling for the imaging department, MDT planning, and the patient diary planning, but cannot determine what the symptoms will be at the point of imaging. The radiologist only has information of symptoms at the time of request, which may limit interpretation. This allows decisions to be made on commencing, continuing, or discontinuing treatment and provides insight into whether treatment has caused a meaningful alteration in tumour biology. Standard structural MRI is routinely used for this purpose. In some centres, “advanced” MRI techniques (e.g. dynamic-susceptibility contrast enhanced, DSC, imaging or 1H-magnetic resonance spectroscopy MRS) or PET (targeting glucose or amino acid uptake) helps problem solving in instances when structural imaging is indeterminate (16, 17). As an alternative to a planned imaging schedule, imaging can also be triggered by a change in symptomatology or clinician concern, regardless of any scheduled follow-up interval. While triggered imaging is more difficult to organise at short notice, the strategy benefits from addressing patient concerns regarding the cause of new symptoms and providing the radiologist with contemporaneous clinical information at the time of imaging. In most centres the strategy for interval imaging is a combination of both a planned schedule and triggered imaging (16). Clinical and other non-imaging biomarkers of disease progression, whether as triggers for imaging or additional treatment response biomarkers, have the potential to be incorporated into the patient pathway and would benefit from further research (**Supplementary Information 1**).

The emergence of novel therapies, such as immunotherapy have created challenges for follow-up imaging of glioma in clinical trials, with pseudophenomena occurring in up to 5% of patients leading to the development of modified response assessment approaches such as iRANO (26). The central modification is that the moratorium on progressive disease is extended to cover the first 6 months of treatment. Stopping routine interval imaging for 6 months is, however, not recommended given the potential for the side effects of these therapies. Immunotherapy is not currently recommended as a second-line treatment option in most countries outside of research trials.

### Interval Imaging and Confounds

Although MRI is a safe and effective technique, structural imaging can lead to false positive, false negative, and indeterminate results, particularly relating to post-treatment related pseudophenomena in glioma. In glioblastoma, pseudoprogression is an early post-treatment related effect typically occurring within 6 month of finishing concomitant temozolomide and radiotherapy whereas pseudoresponse typically occurs after anti-angiogenic agents such as bevacuzimab have been administered. False positive progression and false negative treatment response are manifest as an increase or decrease in MRI contrast enhancing volume respectively. Confounding of treatment response commonly occurs with the use of current standard interval imaging conventions in therapeutic and novel imaging glioma trials due to the impact of such pseudophenomena (27). Delayed treatment effects such as increased enhancement due to radiation necrosis can similarly cause false positive progression. Other examples of non-specificity include post-operative peritumoral parenchymal enhancement following operative “tissue handling”; or following operative infarction. Confounding is particularly relevant if progression-free survival is used as an outcome measure which is fundamentally based on, and therefore affected by, the timing of routine interval imaging. In part to mitigate this, objective criteria such as RANO require a threshold of enhancing size change (a 25% increase or 50% decrease in the product of perpendicular dimensions) and an indication of clinical status, corticosteroid dose, and other possible confounds of deterioration such as unrelated health issues; and for true positive progression there is a requirement for sustained size change beyond one time point. It is noteworthy that overall survival is also confounded by differences in treatment at progression. Management typically consists of second-line chemotherapy including the combination of procarbazine, lomustine and vincristine (PCV) (28–30), TMZ re-challenge (31, 32) or supportive care. Not only is management heterogenous, but pseudophenomona will confound this management choice. Furthermore, such detail to understand these co-variates are rarely included in studies (33).

### The Patient and Carer Experience of Interval Imaging

Patients undergoing MRI often experience anxiety prior to and during scanning (34). Up to 37% of patients undergoing MRI experience moderate to high levels of anxiety related to the procedure itself (35–37). When the patient is aware they have a brain tumour that is being assessed for response or progression, anxiety is likely to be more frequent and magnified. Such “scanxiety” is a recognised consequence of interval imaging in cancer (19). Incorporating phenomena such as “scanxiety” into neuro-oncological studies requires patient-reported outcome (PRO) measures which are well defined and reliable and therefore can generate high-quality evidence (38).

Uncertainty is defined as an individual’s “lack of ability to determine the meaning of illness‐related events” (39). In patients with primary brain tumours this has a direct impact on all negative mood states (tension, depression, anger, fatigue, and confusion) measured using the Profile of Mood States‐Short Form (POMS‐SF)) (40). These negative mood states impact on symptom severity, with higher levels of uncertainty associated with worse negative mood states and symptom severity. Due of the high likelihood of disease progression or recurrence in glioma, negative mood states may be exacerbated when patients who have symptoms are awaiting MRI results. Interventions designed to reduce uncertainty may help lessen patients’ perception of symptom severity, which may subsequently result in better treatment outcomes and quality of life. One solution might be “one-stop” clinics in neuro-oncology, but this can be logistically challenging due to managing scanner capacity and radiologist availability for providing direct access reporting. Another approach to reduce uncertainty might be to provide re-assurance that the disease is better or to give a clear management plan for treatment at the point when the imaging results are conveyed to them. Conversely, any new uncertainty or uncertainty that persists following imaging, such as the consideration of pseudophenomena, might reinforce or perpetuate negative mood states. In the typically slower progressing lower grade glioma and meningioma, where there is less impact of pseudophenomena and delayed treatment effects, it is unclear how the relatively long imaging intervals influence uncertainty.

There are several sources of low-level evidence (level 4) indicating that patient and carer anxiety related to perceived unnecessary MRI scans or inaccurate or indeterminate imaging findings in primary brain tumours is a concern. This was a motivating factor behind the James Lind Alliance Priority Setting Partnership priority to establish the value and benefit of neuro-oncological interval scanning (18). Study design into interval imaging would benefit from including patient-reported outcomes (PRO) so that “uncertainty reduction” can be measured.

### Interval Imaging Practices With a Focus on Glioblastoma

There is no robust evidence (< level 3) to support the value or lack of value for the imaging practices currently used to monitor disease or to determine the response of any treatment given in adult brain tumours (4). There is also a lack of evidence around the utility of early post-operative MRI (EPMRI; within 72 h) on adult brain tumour patients after surgical resection of glioblastoma (16, 41–43). Interval imaging conventions are based predominantly on expert opinion and have been primarily motivated by efforts to standardise outcomes for comparing therapeutic trials (9, 11). For EPMRI, it is also noted that indirect contributors to value, such as improving surgical practice (5, 44), are challenging to measure, particularly at the start of a complex treatment pathway. Any study design into interval imaging value must consider current or planned therapeutic trials where outcomes are based on interval imaging regimens. Similarly, there should be awareness that current imaging conventions and the reliance of regulatory approval pathways on them [e.g. FDA endorsing RANO-based treatment outcomes (45)] might impede the development of innovative imaging solutions and other biomarkers designed to rationalise or optimise the imaging pathway.

An understanding of current practice is critical to subsequent study design. A recent UK-wide national clinical practice survey on the use of interval imaging in glioblastoma management (GIN CUP study) showed considerable variation between centres (16). Similarly, the timing and interval length between MRI examinations in the period following completion of adjuvant chemotherapy, shows considerable inter-institutional variation. It is also noted that current UK, European, and international guidelines (4, 46–49) show variation and lack of consensus on the frequency and timing of neuroimaging during the post treatment follow-up period, likely as a result of the lack of objective evidence base and different resourcing between jurisdictions.

In summary, there is considerable variation in interval imaging practice between centres during the glioblastoma post treatment follow-up period which should be considered in the study design of interval imaging value. Neuroimaging is believed to be crucial in making subsequent plausible management decisions once treatment is initiated, however there is a paucity of evidence for this assertion at all timepoints. Additional evidence, therefore, needs to be obtained to determine whether imaging protocols used in current routine clinical practice, and the type of neuroimaging performed at each component of the pathway, result in a measurable and impactful change in management (as opposed to the perception of a change or impact). Determining whether there is a change in outcome and value (morbidity, mortality, quality of life or resource use) is key.

### Health Economics of Interval Imaging

Economic evaluation addresses issues of efficiency and cost effectiveness: are the resources required to provide the intervention, in this case MRI scans, justified by the health benefits? If the MRI scan offers no health benefit, then the intervention is not considered cost effective, with robust economic evaluation required to determine under which circumstances cost-effectiveness is achieved. Whilst retrospective analyses may determine to some extent whether there has been a change in management, prospective studies are needed to quantify the benefit in the context of confounds.

To determine cost effectiveness, an estimate of the impact of imaging on overall resource use is required as well as an estimate of the impact of imaging on survival and quality of life. Consideration of resource data collection is important in the design of future studies. Prospective collection of quality of life data can support a within-trial analysis of quality adjusted life-years (QALYs: a measure of the impact of the intervention on health-related quality of life and survival). To inform QALYs, quality of life is typically measured using generic quality of life instruments, of which the most commonly used are the EuroQol EQ-5D (50), the SF-6D (51) (based on the Rand SF-36 questionnaire) and the health utilities index (52). The EQ-5D is the most commonly used measure, and reports health status as the level of functioning in five domains. The five level (5L) instrument differentiates 3,125 response combinations each of which has an associated tariff ranging from 1 for full health, through 0 for dead, to negative scores for a small number of health states considered worse than dead. Measurement might be performed at baseline and 3 monthly intervals during the imaging period, in a similar fashion to interval imaging. More extensive questionnaires such as the European Co-operative Oncology Group Quality of Life Questionnaire Core 30 (EORTC QLQ C30) (53) may provide a more targeted capture of quality of life and may prove to be suitable instruments. Further work is required to determine more detailed evidence surrounding patients’ views on quality of life in relation to interval imaging cost effectiveness. Other sources of information to capture outcomes such as mortality or resource use can come from case report forms (CRF) within a trial or from clinical registers, e.g. the Surveillance, Epidemiology and End Results database in the US (SEER) (54) and the National Cancer Registration and Analysis Service (NCRAS) in the UK (55). Administrative databases may also provide useful data on diagnosis, treatments and survival (56). The literature also provides estimates of the cost of care for relevant events that may not be observed during a trial such as the cost of end-of-life-care (EOLC) (57).

Trial follow-up is frequently insufficient to capture the full implications of monitoring and treatment on patient costs and outcomes. A decision model is commonly used to extrapolate costs and outcomes beyond trial follow-up, often over the remaining lifetime of the patient cohort. The most commonly used is the Markov model, which captures patient trajectories as a sequence of health states representing progression of the disease (58). Estimation of lifetime costs and outcomes of different monitoring and treatment strategies allows quantification of the difference in costs and outcomes across strategies. The ratio of incremental costs to incremental outcomes, known as the incremental cost-effectiveness ratio (ICER), reports the efficiency of more effective strategies in terms of the cost per unit improvement in outcome. These data typically influence recommendations from national health technology agencies on the use of new technology and care pathways, although the application of an explicit upper limit or threshold with regard to cost-effectiveness is limited to the UK, Australia and Canada (59).

Deriving evidence to determine the cost effectiveness of interval imaging requires consideration of the impact of imaging on downstream costs and outcomes. Downstream costs for surgical treatments such as craniotomy or licensed chemotherapy drugs can greatly outweigh the costs of the imaging. Hence quantifying small changes in treatments arising from imaging is important. Therefore, any design or modelling to optimise interval imaging cost effectiveness in routine clinical practice should incorporate changes in the costs of any subsequent alteration in treatment i.e. related and opportunity costs. For example, the model should incorporate changes in the costs of continuing expensive and ineffective therapies which themselves may be associated with adverse effects; changes to surgical procedures which themselves may be associated with reduced or prolonged hospital stays; and changes to the costs of rehabilitation if the clinical impacts of progression of underlying disease are altered.

The conclusions of any health economic design framework described above are most applicable to integrated healthcare systems such as the UK. In these healthcare systems, imaging was historically considered relatively costly, and most agencies endorse rationing which can limit use. However, other reimbursement models in other healthcare systems can incentivise additional investigation, as reflected by the wide discrepancy in MRI use between countries (60).

Beyond providers, there are individual financial implications for imaging. For example, 54% of carers of US patients with high-grade gliomas out of active treatment had costs of $271 per month with transportation to hospitals amongst the greatest out of pocket costs (61). These personal costs may be lower in healthcare systems such as the UK where hospitals and charities provide additional support, but evidence suggests they remain substantial (62). Health economic modelling would benefit from incorporating such individual costs and regional/international variations.

More evidence to determine the cost effectiveness of interval imaging incorporating the patient, carer, and healthcare system is required. Careful study design using standard tools should achieve this. Evidence on cost-effectiveness will improve care pathways in all systems, and is central to the efficient use of resources in centrally funded healthcare systems.

### Data Science *In Silico* Interval Imaging Studies

*In silico* studies are those performed on a computer or *via* computer simulation. Sophisticated algorithms or simulations can advance scientific understanding, although the inferences drawn must recognise the limitations introduced by the simplified or reductive framework. The results of these simulations can be tested in existing trials or serve as a guide for future trials. Machine learning applications may move beyond inferential statistical approaches to attempt to extract more accurate predictions from complex datasets. Such approaches for imaging monitoring biomarkers in neuro-oncology are at an early stage of development in terms of clinical validation and applied techniques are not yet ready to be incorporated into the clinic (63, 64). A recent systematic review using PRISMA-DTA and QUADAS-2 methodology, showed that the small numbers of patients included in machine learning studies, the high risk of bias and concerns of applicability in the study designs, and the low level of evidence given that the monitoring biomarker studies are retrospective, suggest that limited conclusions can be drawn from the data (33).

Studies may take advantage of enhanced computational approaches to build data-rich neuro-oncology monitoring biomarker models, although more involved or computationally expensive approaches such as those used in deep learning, may not *de facto* outperform more traditional machine learning techniques, for example multivariate logistic regression (63). It is also notable that studies applying machine learning to build neuro-oncology monitoring biomarker models have yet to show overall advantage over those using traditional statistical methods in terms of analytical validation and diagnostic performance (63, 65, 66). Such statistical methodology is wide ranging and includes generalised estimating equations and mixed models (67) but for clarity, we note that there is a continuum between the two fields, a pertinent example being non-parametric orthogonal transformations for dimensionality reduction.

We note several barriers in translating machine learning which the neuro-oncology community must appreciate for *in silico* study design: (1) the clinical context may not be represented with a decreased ability to perform holistic evaluations of patients, with loss of valuable and irreducible aspects of the human experience such as psychological, relational, social, and organizational issues (68); (2) accuracy-driven performance metrics have led to more opaque models (69) although advances in interpretability and explainability may mitigate this somewhat (70); (3) binding the empirical data to categorical interpretation misses an intrinsic ambiguity in the observed phenomena (71) which might negatively affect performance (68); (4) overreliance on the capabilities of automation can lead to the related phenomenon of deskilling (72). Furthermore, there are several technical limitations that make many algorithms unreliable: domain adaptation is still in its infancy and further solutions are required to help algorithms extrapolate well to new hospitals. Uncertainty estimation is still underdeveloped, and necessary to know when algorithms are out-of-distribution or when the accuracy might be poor. Robustness to data issues, such as artefacts, is very much needed but also at its infancy. Lastly, the presence of multiple pathologies (for example, tumours and stroke) can also confound algorithms as these cases are rare and often unlabelled.

Nonetheless, we emphasise that machine learning models have key advantages: whilst three decades ago it was noted that they require less formal statistical training given developments in software (73, 74), more recently there has also been a transformative reduction in the requisite programming expertise for researchers which has been enabled by open source software standardised implementations (75–77); have the ability to detect implicitly any complex non-linear relationship between independent and dependent variables (73, 78); and have the ability to detect all possible interactions between predictor variables (69). Indeed, new approaches have proven to bring new perspectives and insights to the diagnosis of neuro-oncology pathologies, such as glioma (79, 80). In particular, some of these models are currently used as diagnostic biomarkers (12) for prediction of tumour grading and genomics from imaging as well as automating diagnosis from histopathology; furthermore prognostic biomarkers can provide insights into survival (80).

Advances in brain tumour database curation will facilitate integration of imaging data with demographic, clinical, and molecular marker data into large databases [in the UK, for example, these include Health Data Research UK, the Tessa Jowell BRAIN MATRIX (81) or BRIAN – the Brain tumouR Information and Analysis Network (82)]. The capture of large volumes of data and the inclusion of a wider spectrum of imaging phenotypes, typically results in improved diagnostic performance during machine learning or statistical tasks; the relative improvement of deep learning model performance is particularly marked (83–85). Note that for deep learning, the dependency on very large datasets can be reduced by data augmentation and transfer learning; the latter, where an already developed model for a task is reused as the starting point or a model on a second task, is especially advantageous for medical tasks since these pre-trained models not only obviate the need for very large datasets but are less computationally expensive (70, 79, 80). Once established, incoming data from each of these larger scale live repositories will facilitate ongoing refinement and assessment of impacts. Examples of machine learning tools that have been used with large datasets in neuro-oncology, as well as generic approaches to multi-centre machine learning which might overcome privacy issues, are contained in the **Supplementary Information 2**.

Initiatives and consensus statements have provided recommended frameworks (86–89) for standardising imaging biomarker discovery, analytical validation, and clinical validation (12), which can help to improve the robustness of study design of machine learning applied to neuro-oncology. It is clear that for such an approach large, well-annotated datasets, and therefore, multi-disciplinary and multi-centre collaborations are mandated (63), and this will require a collaborative approach to reach meaningful dataset size and quality.

### Interval Imaging Study Design and Statistical Considerations

The overarching purpose of any study design would be to determine the value of interval imaging and to maximise this value where possible. Ideally, studies would provide robust evidence (≤ level 3) for morbidity, mortality, quality of life and resource use (together these outcomes give the additional outcome measure of cost effectiveness) of three tumour groups (meningiomas, lower grade gliomas, and high-grade gliomas) undergoing interval imaging. Further Patient and Public Involvement (PPI) work is underway to refine measurable metrics although a primary outcome of mortality and secondary outcomes of quality of life e.g. the EUROQOL EQ 5D–5L or EORTC QLQ C30 score, may be sensible. Outcomes are confounded by treatment type and motivate thorough co-variate collection. Progression-free survival is especially confounded as described above and must be considered carefully as an outcome measure in glioma study design. This is a major driver for the consideration of adopting “advanced imaging” in more robustly defining a progression event through imaging.

Given these, it is likely that different approaches are required to construct an evidence framework surrounding interval imaging (**Table 1**); building the framework is likely to be stepwise (90), using less robust evidence (< level 3) initially as well as determining baseline quality of life and resource use outcomes. Whilst the trial giving the highest level of evidence would be a randomised controlled trial (RCT), and likely a non-inferiority design, knowing which aspects of the pathway to randomise will require additional supportive intermediate evidence from preliminary studies. For example, data can be acquired using audit or observational studies to determine whether there is a change in management or not. If management is changed, an RCT may be able to address whether there is additional value from the change in management in terms of morbidity, mortality, quality of life and resource use. However, there may be challenges for recruitment of patients into an RCT, predominantly influenced by tumour type. For example, in a high-grade glioma RCT with reduced imaging in one arm, some participants and recruiters may oppose reduced imaging in a tumour where changes in disease can be rapid. It is plausible that there would be less concern for lower grade gliomas or meningioma interval imaging studies.

*In silico* studies using statistical or machine learning approaches might provide an alternative to inform which aspects of the pathway should be randomised in an RCT (91, 92). Alternatively, such techniques might be used to approximate outcomes themselves, however, as with an RCT, a large number of centres would be required to provide sufficient data, particularly if PROs and health economic measures are also incorporated. It is noteworthy that within existing provision and clinical trials, there will be natural jitter and missed time points in the follow-up of patients. With large datasets this might provide an opportunity using appropriate modelling techniques to assess the impacts of these natural timing differences and missing data points. Despite the potential of *in silico* studies, a disadvantage is that they do not produce level 1 evidence nor is it clear how the most complex modelling studies equate with traditional levels of evidence (3).

Whilst the focus of study design relates to the structural MRI interval imaging pathway and by default the “when” of imaging, the “how” and “what else” remain important avenues for research (5). It is conceivable that the interrogation of biomarkers such as MRI radiomic features, advanced MRI or PET studies can be added as secondary objectives. It is acknowledged that these are not routinely used nor widely available modalities, and in the case of PET in particular, have a distinct risk and cost effectiveness profile compared to structural MRI. We note other expert consortia are looking specifically into advanced imaging and processing techniques to develop international recommendations and guidelines on their application as monitoring biomarkers (93, 94).

Regardless of the approach to achieve accurate, complete, and transparent reporting of studies contributing to the evidence of interval imaging in standard of care brain tumour management, we strongly recommend following reporting guidelines from the EQUATOR Network (95), available for example in prospective biomarker studies (96, 97), RCTs (98) or economic evaluations (99).

## Discussion

Determining the value, and therefore the utility, of interval imaging in brain tumour management remains a key priority in neuro-oncology. Meningioma and glioma were identified as priorities for interval imaging utility analysis. Any study design that changes imaging regimens should consider the potential for influencing current or planned therapeutic trials; ensure opportunity costs are measured; and that indirect contributions to value are identified and assessed.

Whilst it was agreed that an RCT would provide level 1 evidence, no consensus was reached on specific trial design, reflecting the immense challenge faced in addressing this evidence gap. While development of level 1 evidence is the desired goal, given that current practice is predominantly based on expert opinion (level 5) there is a role for establishing “intermediate level” evidence that might support a future RCT. The outcomes of any study must include overall survival, quality of life and resource use. The panel agreed that this “intermediate level” evidence was unlikely to be obtained solely through descriptive and inferential statistics of existing datasets and would benefit from modelling and advanced statistical and machine learning approaches, and that larger, aggregate datasets would be required involving multicentre collaborations. Overall, no consensus was reached as to the specific studies which should be undertaken, but types of study have been described here for consideration along with their strengths and limitations.

This position statement aims to provide a framework for developing the evidence base for the value of interval imaging in primary brain tumours and, thereafter, practice recommendations. The panel welcomes any collaborative approach from groups interested in aggregating data and contributing to study design. Ongoing collaborative efforts will improve trial design and generate the evidence to optimise monitoring imaging biomarkers in standard of care brain tumour management.

## Author Contributions

FB, TB HB, CB, JC, LD, RG, MJ, CMc, SM, NM, CMu, MP, JP, DS, GT andMWcontributed conception and design of the study. TB wrote the first draft of the manuscript. FB, TB, HB, JC, RG, MJ, SM, NM, MP, JP, DS, GT, AW and MW wrote sections of the manuscript. DJ, KA, JK, NH, AL, SO, AW and CW: Not in attendance at incubator day; provided subsequent input. All authors contributed to the article and approved the submitted version.

## Funding

This Interval Imaging Incubator Day meeting was facilitated and funded by *brainstrust* – the brain cancer people. No participants were remunerated for their contribution. National Cancer Research Institute (NCRI) separately provided travel and accommodation for those panel members who had travelled for either the main brain tumour group meeting or the glioma subgroup. This work was supported by the Wellcome/EPSRC Centre for Medical Engineering [WT 203148/Z/16/Z].

## Conflict of Interest

TB, speaker’s bureau for AbbVie and Siemens Healthineers. Craig Buckley, Head of Research and Innovation – Siemens Healthineers GB&I. JC, BrainMiner Founder. Involved in machine learning enterprise and business. JK, involved in enterprise and business. MM, BrainMiner Founder. Involved in machine learning enterprise and business. SO, BrainMiner Founder. Involved in machine learning enterprise and business. MP, received payment for consultancy work from Merck not related to cancer. AW, unrestricted educational grant, Bayer Schering, consultancy work and honoraria not related to cancer.

The remaining authors declare that the research was conducted in the absence of any commercial or financial relationships that could be construed as a potential conflict of interest.
