# Supplementary material for: A Position Statement on the Utility of Interval Imaging in Standard of Care Brain Tumour Management: Defining the Evidence Gap and Opportunities for Future Research
Source: Front Oncol. 2021 Feb 9;11:620070. doi: 10.3389/fonc.2021.620070 (PMC7900557; doi:10.3389/fonc.2021.620070)
Supplement: Supplementary file 1 [file DataSheet_1.pdf]

Supplementary Material

1 **Supplementary Table 1.** The National Institute for Health and Care Excellence Brain Tumour Guideline NG99 showing advantages and disadvantages of imaging follow-up frequency[1].

**Possible advantages of more frequent follow-up**	**Possible disadvantages of more frequent follow-up**
May identify recurrent disease earlier which may increase treatment options or enable treatment before people become symptomatic.	There is no definitive evidence that identifying recurrent disease early improves outcomes.
May help provide information about the course of the illness and prognosis.	May increase anxiety if changes of uncertain significance are detected on imaging.
Some people can find more frequent imaging and hospital contact reassuring.<br>Provides an opportunity to identify patient or carer needs (such as psychosocial support and late side effects of treatment).	Some people can find more frequent imaging and hospital contact burdensome and disruptive – they feel their life revolves around their latest scan.<br>There may be a financial cost from taking time off work and travelling to appointments.
–	More imaging and follow-up is resource intensive for the NHS.

Supplementary Table 2. The National Institute for Health and Care Excellence Brain Tumour Guideline NG99 possible regular clinical review schedule for people with glioma depending on grade of tumour[1]. Currently, there is a variety of schedules within the UK[2].

<table>
<tr><th rowspan="2"></th><th colspan="6">Years after end of treatment</th></tr>
<tr><th>0 to 1</th><th>1 to 2</th><th>2 to 3</th><th>3 to 4</th><th>5 to 10</th><th>>10 (for the rest of life)</th></tr>
<tr><td>Grade I</td><td colspan="6">Scan at 12 months, then:
consider discharge if no tumour visible on imaging unless completely resected pilocytic astrocytoma
consider ongoing imaging at increasing intervals for 15 years for completely resected pilocytic astrocytoma
consider if ongoing imaging is needed at a rate of once every 1 to 3 years for the rest of the person's life if the tumour is visible on imaging.</td></tr>
<tr><td>Grade II 1p/19q non-codeleted, IDH mutated</td><td colspan="2" rowspan="3">Scan at 3 months, then every 6 months</td><td colspan="2" rowspan="3">Annually</td><td rowspan="3">Every 1 to 2 years</td><td rowspan="3">Consider ongoing imaging every 1 to 2 years</td></tr>
<tr><td>Grade II 1p/19q codeleted</td></tr>
<tr><td>Grade III 1p/19q codeleted</td></tr>
<tr><td>Grade II IDH wildtype</td><td colspan="2" rowspan="3">Every 3 to 6 months</td><td colspan="2" rowspan="3">Every 6 to 12 months</td><td rowspan="3">Annually</td><td rowspan="3">Consider ongoing imaging every 1 to 2 years</td></tr>
<tr><td>Grade III 1p/19q non-codeleted</td></tr>
<tr><td>Grade IV (glioblastoma)</td></tr>
</table>

3 **Supplementary Table 3.** The National Institute for Health and Care Excellence Brain Tumour Guideline NG99 possible regular clinical review schedule for people with meningioma depending on grade of tumour[1].

	**Years after end of treatment**									
	**0 to 1**	**1 to 2**	**2 to 3**	**3 to 4**	**4 to 5**	**5 to 6**	**6 to 7**	**7 to 8**	**8 to 9**	**>9 (for the rest of life)**
**Grade I: no residual tumour***	Scan at 3 months	Annually		Once every 2 years						Consider discharge
**Grade I: residual tumour***	Scan at 3 months	Annually				Once every 2 years				Consider discharge
**Grade I: after radiotherapy**	Scan 6 months after radiotherapy	Annually		Once every 2 years						Consider discharge
**Grade II**	Scan at 3 months, then 6 to 12 months later	Annually				Once every 2 years				Consider discharge
**Grade III**	Every 3 to 6 months		Every 6 to 12 months			Annually				
**Asymptomatic incidental meningioma**	Scan at 12 months. If no change consider discharge or scan at 5 years.									

* The presence of any residual tumour can only be established after the first scan at 3 months.

4 Supplementary Information 1

Apart from change detected on imaging, there are other known clinical predictors of disease progression or patient survival, including cognitive decline[3-5], depressive symptoms[6], deterioration of health-related quality of life after surgery[7], and instrumental activities of daily living in older patients[8]. Although some sort of regular clinical review is recommended[1], unlike imaging, these predictors are not currently formally assessed at regular intervals nor is there unity in choice of index test. A key challenge will be to transform promising prognostic biomarkers into monitoring biomarkers. Exemplars would be the two randomised controlled trials conducted in patients with lung [9] and metastatic solid (breast, lung, genitourinary, gynaecological) cancer[10], respectively, demonstrating that the systematic real time reporting of patient-recorded outcomes during routine surveillance can improve health-related quality of life, and overall survival.

There has been promising research into the potential value of circulating biomarkers (for example circulating tumour cells, exosomes, and microRNAs) to monitor disease progression in glioma patients [11-16]. A recent study found that serum micro-RNA levels did not increase in cases of pseudo-progression and increased levels were associated with disease progression[14]. However, as with any potential monitoring blood or cerebral spinal fluid biomarker, potential use requires further evaluation and validation in larger scale prospective studies before implementation into routine clinical practice can be envisaged.

5 Supplementary Information 2

To achieve the potential of robust *in silico* studies designed to determine whether interval imaging is valuable and to maximise this value where possible, tools must be used that are fit-for-purpose. These include, but are not limited to, The Extensible Neuroimaging Archive Toolkit (XNAT) for data curation (*https://www.xnat.org/),* Cogstack for Electronic Health Record (EHR) extraction (*https://ctiuk.org/products/cogstack/)* and NiftyNet for neuroimaging processing (*https://niftynet.io/).* Improvements to deep learning neuroimaging models to specifically incorporate non-imaging information are being developed[17], as are methods to counter the lack of counterfactual information[18].

Studies involving multiple sites will benefit from data repositories in single sites undergoing irreversible data anonymisation during export to a centralised secure sandbox (a virtual space in which new or untested software or coding can be run securely) to allow distributed learning[19,20]. A specific distributed machine learning approach, federated learning, enables training on a large body of decentralized data[21]. Federated learning is one instance of the more general approach of "bringing the code to the data, instead of the data to the code" and addresses the fundamental problems of privacy, ownership, and locality of data. Whilst this technique is at the research stage, federated learning appears to be fit-for-purpose for privacy preserving medical applications[22,23], and specifically for brain tumour interval imaging *in silico* studies. However, the potential privacy and performance trade-off is unknown. Once established, federated learning will likely speed up the validation of the proposed methods, since there will be less administrative data access requirements yet the sample will continue to be enlarged by new data from several sites.

6 References

[1] The National Institute for Health and Care Excellence. Guideline NG99. https://www.nice.org.uk/guidance/ng99. [Accessed 1 March 2019].

[2] Booth TC, Luis A, Brazil L, Thompson G, Daniel RA Shuaib H et al. Glioblastoma post-operative Imaging in Neuro-oncology: Current UK practice (GIN CUP study) Eur Radiol (2020) https://doi.org/10.1007/s00330-020-07387-3

[3] Meyers CA, Hess KR, Yung WK, Levin VA. Cognitive function as a predictor of survival in patients with recurrent malignant glioma. J Clin Oncol. (2000) 18(3):646-50.

[4] Butterbrod E, Synhaeve N, Rutten GJ, Schwabe I, Gehring K, Sitskoorn M. Cognitive impairment three months after surgery is an independent predictor of survival time in glioblastoma patients. J Neurooncol. (2020) 149(1):103-111

[5] Lee ST, Park CK, Kim JW, Park MJ, Lee H, Lim JA, et al. Early cognitive function tests predict early progression in glioblastoma. Neurooncol Pract. (2015) 2(3):137-143.

[6] Noll KR, Sullaway CM, Wefel JS. Depressive symptoms and executive function in relation to survival in patients with glioblastoma. J Neurooncol. (2019) 142(1):183-191.

[7] Jakola AS, Gulati S, Weber C, Unsgård G, Solheim O. Postoperative deterioration in health related quality of life as predictor for survival in patients with glioblastoma: a prospective study. PLoS One. (2011) 6(12):e28592. doi: 10.1371/journal.pone.0028592.

[8] Lorimer CF, Walsh G, MacKinnon M, Corbett A, Bedborough K, Greenwood K, et al. Geriatric assessment of glioblastoma patients is feasible and may provide useful prognostic information. Neurooncol Pract. (2020) 7(2):176-184.

[9] Denis, F., et al., Two-Year Survival Comparing Web-Based Symptom Monitoring vs Routine Surveillance Following Treatment for Lung Cancer. JAMA. (2019) 321(3):306-307

[10] Basch, E., et al., Symptom Monitoring With Patient-Reported Outcomes During Routine Cancer Treatment: A Randomized Controlled Trial. J Clin Oncol (2016) 34(6):557-65.

[11] Hermansen, S.K. and B.W. Kristensen, MicroRNA biomarkers in glioblastoma. J Neurooncol (2013) 114(1): 13-23.

[12] Ma C, Nguyen HPT, Luwor RB, Stylli SS, Gogos A, Paradiso L, et al., A comprehensive meta-analysis of circulation miRNAs in glioma as potential diagnostic biomarker. PLoS One (2018) 13(2):e0189452.

[13] Møller HG, Rasmussen AP, Andersen HH, Johnsen KB, Henriksen M, Duroux M.A systematic review of microRNA in glioblastoma multiforme: micro-modulators in the mesenchymal mode of migration and invasion. Mol Neurobiol. (2013) 47(1):131-44.

[14] Morokoff A, Jones J, Nguyen H, Ma C, Lasocki A, Gaillard F et al., Serum microRNA is a biomarker for post-operative monitoring in glioma. J Neurooncol (2020) 149(3):391-400.

[15] Zhi, F., et al., Identification of 9 serum microRNAs as potential noninvasive biomarkers of human astrocytoma. Neuro Oncol, 2015. 17(3):383-91.

[16] Müller Bark J, Kulasinghe A, Chua B, Day BW, Punyadeera C. Circulating biomarkers in patients with glioblastoma. Br J Cancer (2020). 122(3):295-305.

[17] Wood D, Cole J, Booth TC. NEURO-DRAM: a 3D recurrent visual attention model for interpretable neuroimaging classification. [Preprint] (2019). Available at: https://arxiv.org/abs/1910.04721 [Accessed 1 December 2019]

[18] Atan O, Zame WR, Feng Q, van der Schaar M. Constructing Effective Personalized Policies Using Counterfactual Inference from Biased Data Sets with Many Features. [Preprint] (2018) https://arxiv.org/pdf/1612.08082.pdf [Accessed 1 December 2019]

[19] Dean J, Corrado G, Monga R, Chen K, Devin M, Mao M et al. Large scale distributed deep networks. In: Pereira F et al. editors. Advances in Neural Information Processing Systems 25. (2012) p. 1223–1231.

[20] Low Y, Bickson D, Gonzalez J, Guestrin C, Kyrola A, Hellestein JM. Distributed graphlab: A framework for machine learning and data mining in the cloud. Proc VLDB Endow. (2012) 5:716–727.
[21] Bonawitz K, Ivanov V, Kreuter B, Marcedone A, McMahen HB, Patel S et al. owards Federated Learning at Scale: System Design. [Preprint] (2019) https://arxiv.org/pdf/1902.01046.pdf. [Accessed 1 December 2019]
[22] Brisimi TS, Chen R, Mela T, Olshevesky A, Paschalidis IC, Shi W et al. Federated learning of predictive models from federated electronic health records. Int J Med Inform (2018) 112:59–67.
[23] Li W, Milletari F, Xu D, Rieke N, Hancox J, Zhu W et al. Privacy-preserving Federated Brain Tumour Segmentation [Preprint] https://arxiv.org/abs/1910.00962) [Accessed 1 December 2019]
